# Follicular bronchiolitis and lymphocytic interstitial pneumonia in a Japanese man

**DOI:** 10.1186/1746-1596-6-85

**Published:** 2011-09-21

**Authors:** Tadashi Terada

**Affiliations:** 1 Department of Pathology, Shizuoka City Shimizu Hospital, Shizuoka, Japan

**Keywords:** lung, follicular bronchitis, lymphocytic interstitial pneumonia, histopathology, immunohistochemistry

## Abstract

A 44-year-old Japanese man consulted to our hospital because of cough and sputum. Chest-XP and CT revealed diffuse reticular opacities in both lungs.

A transbronchial lung biopsy (TBLB) showed a moderate infiltration of lymphocytes in the alveolar septae. He was diagnosed as interstitial pneumonia, and treated by drugs. One year later, his condition deteriolated, and a large open biopsy was performed. It showed a diffuse severe infiltration of lymphocytes in the alveolar walls and a few epithelioid granulomas. No bronchiolitis was seen.

Immunohistochemical study denied lymphocyte monoclonality, and he was diagnosed as lymphocytic interstitial pneumonia (LIP). He was treated by steroid.

Six months later, TBLB showed peribronchial lymphocyte infiltration. A large open biopsy also revealed a severe lymphocytic infiltration around the bronchioles, sparing alveolar wall lymphocytic infiltration. Immunohistochemical study denied malignant lymphoma. He was diagnosed as follicular bronchiolitis (FB). One year later, TBLB showed little lymphocytic infiltration in the alveolar walls as well as peribronchial walls. Two years later, his condition became worse, and TBLB showed features of LIP. Later, his condition was stationary for 6 years with mild lung opacities for 6 years. These findings show that LIP and FB are interchangeable and overlapping, and suggest that LIP and FB belong to the same spectrum of benign lymphoproliferative disorders of the lungs.

## Introduction

Follicular bronchiolitis (FB) is a benign lymphoproliferative lung disease characterized by hyperplastic mucosa-associated lymphoid tissue present around the peribronchial spaces [1]. Patients with FB are often associated with collagen vasculitis diseases, immunodeficiency state, hyperimmune state, and hereditary factors. Idiopathic FB is rare. Lymphocytic interstitial pneumonia (LIP) is also a benign lymphoproliferative lung disease characterized by severe lymphocytic infiltration of the alveolar septae [1]. Patients with LIP are also often associated with collagen vascular diseases, immunological diseases, immunodeficiency diseases, lung infections, and drug induced diseases. When FB and LIP are pathologically diagnosed, exclusion of malignant lymphoma is mandatory [1]. Herein reported is a case with benign lung lymphoproliferative diseases with 11 years follow-up.

## Case report

**Figure 1:**
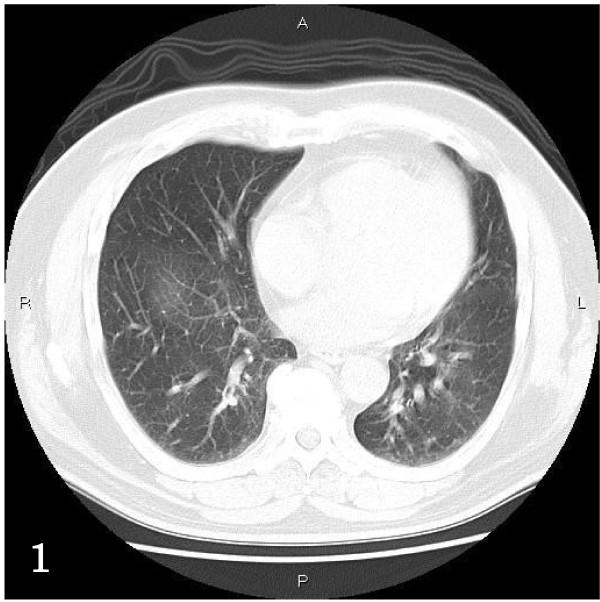
**Chest CT**. Diffuse reticular opacities are seen in both lungs.

**Figure 2:**
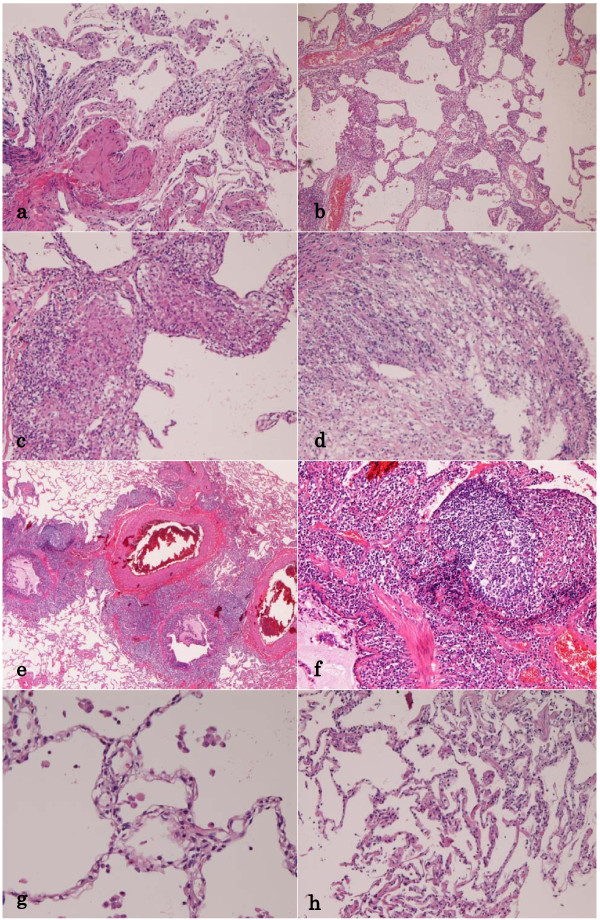
**Lung Biopsies**. (A)The first transbronchial lung biopsy (TBLB). It shows infiltration of lymphocytes in the alveolar septae. HE, ×200. (B)The first large open biopsy. It shows severe lymphocyte infiltration in the alveolar septae. HE, ×100. (C)The first open biopsy. Higher power view. The alveolar septae with severe lymphocytic infiltration and granuloma formation are seen. HE, ×200. (D)TBLB shows peribronchial lymphocytic infiltration. HE, ×200. (E)The second large open biopsy. Heavy infiltration of lymphocytes is seen around the bronchiole (MALT hyperplasia). Germinal centers are recognized. The alveolar septae are free from significant changes. HE, ×40. (F) The second open biosy. High power view. The peribronchial lymphoid tissue has germinal center, and consists of mature lymphocytes. HE, ×200. (G)TBLB shows no or minimal lymphocytic infiltration in the alveolar septae. HE, ×100. (H)TBLB shows lymphocytic infiltration in the alveolar septae. HE, ×40.

A 44-year-old Japanese man consulted to our hospital because of cough and sputum. Chest-XP and CT revealed diffuse reticular opacities in both lungs (Figure 1). He had no other diseases including collagen vascular diseases, immunological diseases, immunodeficiency diseases, and hypersencitivity disorders. A transbronchial lung biopsy (TBLB) showed a moderate infiltration of lymphocytes in the alveolar septae (Figure 2a). He was diagnosed as interstitial pneumonia, and treated by drugs. One year later, his condition deteriolated, and a large open biopsy (video-assisted thracostomic biopsy) was performed. It showed a diffuse severe infiltration of lymphocytes in the alveolar walls and a few epithelioid granulomas (Figures 2b and 2c). The lymphocytes were free from significant atypia. No bronchiolitis was seen. An immunohistochemical study was performed by Dako's envision method (Dako, Glostrup, Denmark) as previously described [2]. The immunohistochemical study showed that the lymphocytes were positive CD3, CD20, CD45, CD45RO, CD79α, κ-chain, λ-chain. Ki-67 labeling was 10%. The light chain restriction was absent, indicating that the lymphocytes were polyclonal. The lymphocytes were negative for p53 protein. Therefore, the immunohistochemistry denied lymphocyte monoclonality and therefore malignant lymphoma. He was diagnosed as LIP. He was treated by steroid.

Six months later, a TBLB showed peribronchial lymphocyte infiltration (Figure 2d). A large open biopsy also revealed severe lymphocytic proliferation around the bronchioles, sparing alveolar wall lymphocytic infiltration (Figures 2e and 2f). The lymphocytes did not show significant atypia. Germinal centers were scattered. The Immunohistochemical study was the same as the previous study and denied lymphocytic monoclonality (malignant lymphoma). Therefore, the lymphoid proliferation was thought to be mucosa-associated lymphoid hyperplasia. He was diagnosed as FB. One year later, TBLB showed little lymphocytic infiltration in the alveolar walls as well as peribronchial walls (Figure 2g). Two years later, his condition became worse, and a TBLB showed features of LIP (Figure 2h). Later, his condition was stationary for 6 years with mild lung opacities.

## Discussion

The author followed up a patient with lymphoproliferative disease of the lung for 11 years. In the two open lung biopsies, the lymphocytes appeared mature, and immunohistochemical studies showed that the lymphocytes had phenotypes of both B- and T cells. Light chain restriction was absent. P53 was negative, and Ki-67 labeling was low. Therefore, the current case was not malignant lymphoma including extranodal marginal B-cell lymphoma (MALT lymphoma). The patient condition was relatively even for 11 years, suggesting that the lung lesion was not malignant lymphoma.

Thus, the present case is benign lymphoproliferative disease. Benign lymphoproliferative diseases were classified into intrapulmonary lymph nodes, FB, LIP, nodular lymphoid hyperplasia (NLH), and Castleman's disease [1]. The current case is obviously different from intrapulmonary lymph nodes and Castleman's disease (giant lymphoid hyperplasia). The present case is different from NLH, in which larger lymphoid nodules (0.6 cm -6 cm, mean 2.1 cm) were scattered in the lungs [1].

The first open biopsy of the present study is obviously LIP [1]. It is not sarcoidosis, because LIP may show epithelioid granuloma [1]. The second open biopsy was obviously FB [1]. The last TBLB is suggestive for LIP. Therefore, the present case showed LIP →FB→ LIP histologies. The present patient did not show other diseases including collagen vascular diseases. Therefore, the FB and LIP in the present case were idiopathic, a very rare phenomenon. These findings show that LIP and FB are interchangeable and overlapping, and suggest that LIP and FB belong to the same spectrum of benign lymphoproliferative disorders of the lungs. Similar suggestion was reported elsewhere [3].

## Consent

Written informed consent was obtained from the patient for publication of this case report and accompanying images.

## Competing interests

The authors declare that they have no competing interests.
